# Plant Carotenoids as Pigment Sources in Laying Hen Diets: Effect on Yolk Color, Carotenoid Content, Oxidative Stability and Sensory Properties of Eggs

**DOI:** 10.3390/foods10040721

**Published:** 2021-03-29

**Authors:** Kristina Kljak, Klaudija Carović-Stanko, Ivica Kos, Zlatko Janječić, Goran Kiš, Marija Duvnjak, Toni Safner, Dalibor Bedeković

**Affiliations:** 1 Faculty of Agriculture, University of Zagreb, Svetošimunska cesta 25, 10000 Zagreb, Croatia; kkljak@agr.hr (K.K.); ikos@agr.hr (I.K.); zjanjecic@agr.hr (Z.J.); kis@agr.hr (G.K.); mduvnjak@agr.hr (M.D.); tsafner@agr.hr (T.S.); dbedekovic@agr.hr (D.B.); 2 Centre of Excellence for Biodiversity and Molecular Plant Breeding (CoE CroP-BioDiv), Svetošimunska cesta 25, 10000 Zagreb, Croatia

**Keywords:** TETRA-SL hens, marigold, calendula, dandelion, basil

## Abstract

The aim of this study was to evaluate the effect of a supplementation diet for hens consisting of dried basil herb and flowers of calendula and dandelion for color, carotenoid content, iron-induced oxidative stability, and sensory properties of egg yolk compared with commercial pigment (control) and marigold flower. The plant parts were supplemented in diets at two levels: 1% and 3%. In response to dietary content, yolks from all diets differed in carotenoid profile (*p* < 0.001). The 3% supplementation level resulted in a similar total carotenoid content as the control (21.25 vs. 21.79 μg/g), but by 3-fold lower compared to the 3% marigold (66.95 μg/g). The tested plants did not achieve yolk color fan values as the control (13.47) or 3% marigold (11.47), and among them, calendula had the highest values (9.73). Despite the low carotenoid content in diets supplemented with basil herb, iron-induced malondialdehyde (MDA) concentration was low as for marigold (on average 106.83 vs. 92.68 ng/g after 250 min). The treatments differed in sensory color scores for fresh and hard-boiled yolks and flavor while other sensory properties were similar. In conclusion, the supplementation of plants in a hen diet may result in yolks containing carotenoids and other compounds showing a high antioxidant effect.

## 1. Introduction

The composition of egg yolk can be influenced by the diet of the laying hen, which is why eggs are being studied as a functional food; eggs that have been developed have been fortified with selenium, vitamins E, D, B12, and folic acid, and n-3 fatty acids [1]. However, eggs are important natural sources of carotenoids in the human diet, especially the xanthophylls lutein and zeaxanthin due to their role in preventing and reducing cataracts and age-related macular degeneration [2,3]. Carotenoids are also responsible for egg yolk color, as a dietary response depending on the content, type, and ratio of carotenoids [4,5,6]. Color is one of the most important factors in consumer perception of food quality; when considering eggs, yolk color is one of the most important characteristics, along with shell color, taste, and appearance, that influence consumer behavior, preferences, and preferences for eggs [7].

To achieve the desired egg yolk coloration, especially when low carotenoid feeds are used, the hens’ diet is supplemented with various sources of carotenoids. In addition to lutein and zeaxanthin, which are common in feeds in hens’ diets, there are carotenoids that are not common but whose supplementation will achieve enhanced coloration at a lower cost. Canthaxanthin is one of these carotenoids that is widely used in poultry diets, and a small addition of this carotenoid to the yellow carotenoids results in satisfactory egg yolk color [6]. Canthaxanthin and other red carotenoids approved by European Commission [8] for use in poultry diets were selected for their coloring effect; nearly 37% to 50% of the ingested canthaxanthin consumed is deposited in the yolk [9]. In comparison, only 1% of dietary β-carotene, between 4.4% and 23% of dietary lutein, and 23% of dietary zeaxanthin are deposited in egg yolk [9].

Although it is generally assumed that higher yolk coloration intensity implies higher carotenoid content, the addition of red carotenoids such as canthaxanthin in small amounts to achieve enhanced coloration contradicts this widely accepted opinion. Thus, paler egg yolks might have more carotenoids than those with enhanced coloration, i.e., a higher score on the DSM Yolk Color Fan (YCF) scale. Additionally, as a potential skin and eye irritant [10], canthaxanthin poses a health risk to humans. There is growing concern among consumers about the use of synthetic additives in animal nutrition and welfare, and demand for organic eggs is increasing. However, hens in organic farming are fed feedstuffs from natural origin, and artificial yolk pigments such as β-apo-8-carotenoic acid ethyl ester or synthetic canthaxanthin are not allowed.

As a result of factors mentioned above, natural pigments are widely studied and the use of some of them, such as the extract of marigold flower (*Tagetes erecta* L.), is commercially recognized in the poultry industry [5,6,11]. Besides the lutein-rich marigold [12], other flowers accumulating various carotenoids in the petals could also be used as pigment sources. Calendula flowers (*Calendula officinalis* L.), containing the major carotenoids flavoxanthin, lutein, rubixanthin, β- and γ-carotene and lycopene, contain 1110–2760 µg total carotenoids/g fresh flowers [13], while dandelion flowers (*Taraxacum officinale* Weber ex Wigg.) are a source of the carotenoid lutein epoxide called taraxanthin [14]. In addition, green leafy parts of plants could also provide carotenoids for egg yolk pigmentation; basil (*Ocimum basilicum* L.) contains 36.5–82.7 µg of lutein, 33.7–73.3 µg of β-carotene, and 2.3–6.2 µg of zeaxanthin in g of fresh leaves, depending on the variety [15].

Basil herb and flowers of calendula and dandelion have been less studied as sources of pigment for egg yolk coloration compared to marigold. These plant parts also have high antioxidant activity [16,17,18], suggesting that hen diet supplementation could protect fatty acids in egg yolks and thus affect not only color and carotenoid content but also oxidative stability and sensory properties. Thus, they could be a suitable alternative to improve egg quality and provide beneficial bioactive compounds in human diet. Previous studies focused on each aspect of egg quality evaluation separately [5,6,19,20,21,22] rather than on the overall effect of the supplemented pigment source. Therefore, the aim of this study was to evaluate the effect of hen diet supplementation with dried basil herb and flowers of calendula and dandelion and on the color, carotenoid content, iron-induced oxidative stability and sensory properties of egg yolks in comparison to the widely studied marigold or commonly used commercial pigment.

## 2. Materials and Methods

The animal experiment was conducted in accordance with the Croatian directives (Animal Protection Act, OG 102/17, and Regulation on the Protection of Animals Used for Scientific Purposes, OG 55/13), which correspond to the European guidelines for the care and use of animals used for scientific purposes. The animal procedures used in this study were approved by the Bioethics Committee for the protection and welfare of animals at the University of Zagreb Faculty of Agriculture (KLASA 114-04/19-03/01, URBROJ 251-71-29-02/11-19-2).

### 2.1. Hens, Housing and Experimental Diets

A total of 135 TETRA-SL 22-week-old laying hens were by three randomly allotted to one of 45 metal battery cages with 750 cm^2^ per hen. Diets and water for hens were provided ad libitum. Throughout the experimental period, the room temperature was 22 ± 3 °C, and the light period consisted of 16 h light per day.

Upon allocating hens to the cages, a 2-week depletion period when all hens were fed a standard diet without added pigment started. The diet was formulated to meet energy and nutrient requirements of laying hens [23]; feed ingredients and calculated composition are presented in Table 1. The cages were then assigned to nine dietary treatments in a complete randomized design with five repetitions per treatment, and the experimental period lasted 28 days. The same standard diet was used as a base to which 1% or 3% of ground dried flower or herb was added for eight experimental diets whereas control diet contained commercially available pigment Carophyll Red containing canthaxanthin (DSM, Heerlen, Netherlands) in a concentration of 8 mg/kg.

Calendula (*Calendula officinalis* L.), marigold (*Tagetes erecta* L.), and basil (*Ocimum basilicum* L. ‘Genovese’) were produced on the experimental field of the Department of Seed Science and Technology, University of Zagreb Faculty of Agriculture while the dandelion (*Taraxacum officinale* Weber ex Wigg.) flowers were collected on the lawn around the experimental field. The technology used for cultivation followed the usual working pattern for the production of medicinal and aromatic plants. After collection, the flowers of calendula, marigold, and dandelion, as well as the basil herb, were dried at 35 °C for 48 h and ground to pass 1 mm sieve (Cyclotec 1093, Foss Tocator, Hoganas, Sweden). Experimental diets were: B1 and B3, supplemented with 1% and 3% basil herb; C1 and C3, supplemented with 1% and 3% calendula flower; M1 and M3, supplemented with 1% and 3% marigold flower; and D1 and D3, supplemented with 1 and 3% dandelion flowers, respectively.

### 2.2. Sample Collection

During the experimental period, eggs collected every three days were analyzed to determine the stabilization of total carotenoid content in the yolk. Carotenoid content stabilized after the 18th day of the experimental period, after which eggs were collected for all analyses between the 22nd and 28th day. All eggs collected on the 22nd and 23rd day were used for color determination, on the 24th day for carotenoid analysis, on the 25th day for evaluation of egg susceptibility to iron-induced lipid oxidation, and on the three subsequent days at the end of the experimental period for sensory analysis. For color determination using YCF scale and carotenoid analysis, each yolk from the same cage was analysed and average value was taken, giving n = 10 for color and n = 5 per treatment for carotenoid content. For color determination using chromameter and susceptibility to iron-induced lipid oxidation, the collected yolks from each cage were combined for analyses giving n = 5 per treatment for each analysis. For sensory analysis, eggs from the same treatment were mixed and randomly selected yolks were offered to the subjects giving n of 60 to 65 per treatment.

All eggs were analyzed in the shortest possible time and stored at 4 °C when necessary. Eggs collected for color, carotenoid, and iron-induced lipid oxidation analyzes were cracked immediately before analysis; yolks were separated from albumen and dried on a paper napkin. Diet samples were collected at the beginning of the experimental period and stored at −20 °C until carotenoid analysis. Samples were ground to pass a 0.5 mm sieve (Cyclotec 1093, Foss Tocator, Hoganas, Sweden) before analysis, and dry matter content was determined by drying 3 g of each sample 4 h at 103 °C according to the method ISO 6496:1999 [24].

### 2.3. Color Determination

Yolk color was analyzed with both YCF (Egg Multi Tester EMT-5200, Robotmation Co. Ltd., Tokyo, Japan) and Minolta Chroma Meter CR-410 (Minolta Co. Ltd., Osaka, Japan) using the CIE (Commission Internationale d’Eclairage) Lab scale. The L*, a*, and b* values reflect brightness (0 = black, 100 = white), redness (−a = green, a = red), and yellowness (−b = blue, b = yellow), respectively. The instrument was calibrated daily against a standard white plate with characteristics Y = 94.5, x = 0.3158 and y = 0.3323. The color of each yolk was first determined using the YCF scale. Then, the egg yolks from each cage collected on the same day were combined and mixed, and the color was determined using the CIE Lab scale.

### 2.4. Carotenoid Analysis

The changes in carotenoid content in egg yolk during the experimental period were determined using the spectrophotometric method described by Surai et al. [25]. Briefly, carotenoids were extracted with hexane after the egg yolk samples were homogenized with 2 mL of 5% NaCl aq. solution/ethanol (1:1) and combined in a 10 mL volumetric flask and diluted to volume with hexane. The spectrum of the hexane extracts was measured between 400 and 500 nm (Helios γ, Thermo Electron Corporation, UK). The absorbance at the maximum was used, and the total carotenoid content was calculated as β-carotene equivalents (μg/g) using the β-carotene solutions with concentrations ranging from 0.2 to 2.5 mg/L.

A more specific carotenoid analysis, both in diets and in the yolks collected on 24th day of the experimental period, was performed according to AOAC method 970.64 [26]. After saponification with 40% methanolic KOH, carotenoids were extracted with a solution of hexane/acetone/absolute ethanol/toluene (10:7:6:7, v/v/v/v), and 7 mL of the extract was pipetted onto a chromatographic tube with adsorbent connected to a vacuum filtration device. Total carotenes were collected upon elution with hexane/acetone (96:4, v/v) on adsorbent 1 (silica gel G/Hyflo Super-Cel, 1:1, w/w). Using the same adsorbent, monohydroxy and dihydroxy pigments were eluted with hexane/acetone (9:1, v/v) and hexane/acetone (8:2, v/v). Total xanthophylls were eluted with hexane/acetone/methanol (80:10:10, v/v/v) on adsorbent 2 (activated magnesia/Hyflo Super-Cel, 1:1, w/w); polyoxy pigments were calculated as a difference between total xanthophylls and monohydroxy and dihydroxy pigments. The absorbance of carotene fraction was measured at 436 nm while 474 nm was used for all xanthophyll fractions. The concentration of carotenoids in each fraction was calculated using equations given in the method [26], while total carotenoids (TC) were calculated as a sum of carotenoid fractions.

### 2.5. Iron-Induced Lipid Oxidation of Egg Yolks

The susceptibility of eggs to iron-induced lipid oxidation was evaluated using the method described by Kornbrust and Mavis [27]. An aliquot of the mixture of three egg yolks collected per cage and 1.15% KCl was mixed with 80 mM TRIS-malate buffer pH 7.4, 5 mM FeSO_4_ × 7 H_2_O and 2 mM ascorbic acid; the prepared mixture was thoroughly mixed and incubated at 37 °C for 50, 100, 150, 200 and 250 min. At the specified times, 2-mL aliquots were immediately subjected to the malondialdehyde (MDA) assay described by Botsoglou et al. [28]. The MDA concentration in the incubated tubes was calculated from a standard calibration curve prepared using 1,1,3,3-tetramethoxypropane as the precursor of MDA.

### 2.6. Sensory Assessment

Sensory analysis was conducted using a hedonic test with 97 untrained consumers among students and faculty staff; the basic sociodemographic characteristics of the set are presented in Table 2.

Immediately before sensory evaluation, eggs were boiled (hard-boiled) for 12 min and kept warm in water at approximately 40 °C. Before serving, the eggs were peeled, the whites were removed, and the yolks were cut into quarters. One quarter of the yolk was served in odorless paper cups labeled with three-digit number. The order of presentation was defined as a balanced incomplete block design and randomized where each panelist received six samples from nine possible treatments. Five sensory characteristics were evaluated: color, aroma, flavor, texture, and overall acceptability. Each attribute was scored on a structured 10-point scale, where 0 meant “extremely disliked” and 9 meant “extremely liked”. Subjects were placed in separate booths and instructed to use tap water and unsalted bread as palate cleansers prior to each sample.

### 2.7. Statistical Analysis

The effect of treatment on the content of all carotenoids was tested using the Kruskal–Wallis test in agricolae package [29] for R [30]. The *p*-values of the post-hoc test were adjusted using Bonferroni correction, with alpha set at *p* = 0.05. The effects of treatment, time, and their interaction on the change in MDA concentration were analyzed in R software using repeated measurements ANOVA. This method includes the analysis of effects between treatments as well as within-treatment variations over time. Visual inspection of scatter plots of fitted values vs. residuals was used to assess the model for normal distribution of the residuals and variance homogeneity. Means of levels of predictors that were statistically significant at the alpha level of *p* = 0.05 were compared using the Bonferroni correction with alpha set at *p* = 0.05 and the R package lsmeans [31].

For sensory analysis data, the NPAR1WAY procedure of SAS Studio University Edition 3.4 (SAS Institute, 2015) was used for nonparametric analysis with Kruskal-Wallis test and DSCF (Dwas, Steel, Critchlow-Flinger) method for treatment comparison at *p* = 0.05 level.

## 3. Results

### 3.1. Carotenoid Content in Diets and Egg Yolks

The content of carotenoids in the experimental and control diets is given in Table 3 while contents of the plant parts used as pigment sources are given in Supplementary Table S1. The control diet with commercial pigment contained 16.48 µg/g DM of total carotenoids. The addition of calendula, dandelion, and marigold increased the content of total carotenoids by up to 20-fold, compared to the control diet (*p* < 0.001), with marigold being the most effective in increasing the carotenoid content in the diet. In contrast, the diet containing basil had lower (1% supplementation) or slightly higher (3% supplementation) content compared to the control diet (*p* < 0.0001). Dihydroxy carotenoids were the fraction with the highest content in all diets except those supplemented with dandelion. The most extreme example was the diet containing marigold with an average content of 94% of this fraction; this content was up to 8 or 15 times higher than in other supplemented diets. In diets supplemented with marigold and basil, dihidroxy carotenoids accounted for majority of xanthophylls, whereas in diets supplemented with calendula and dandelion this contribution decreased due to the higher monohydroxy and poloxy carotenoid content. In addition, supplementation with calendula and dandelion increased the content of total carotenes, and these two diets contained a fraction of polioxy carotenoids, the content of which was more than twice as high in the diets with dandelion as in those with calendula.

Hen diet supplementation with the tested plants affected the yolk’s carotenoid content, but the ratio of carotenoid fractions between dietary treatments was not similar to one of the diets for all plant supplements (Table 3). Egg yolks from hens fed the control diet had the highest content of monohydroxy carotenoids among the diets (44% of total carotenoids), consistent with the high content in the diet. However, this was not the case for hens fed diets that were supplemented with calendula and dandelion; regardless of the high content of this fraction in the diet, eggs had an average of 2.17 µg/g at 1% and 3.58 µg/g at 3% supplementation. Moreover, the total carotene content in the yolks of all dietary treatments was similarly low, regardless of the high content in the diets, and xanthophylls accounted for 88–92% of the total carotenoids in the yolk. Dihydroxy carotenoids were the fraction with the highest content in the yolks of all diets, including those supplemented with dandelion, in contrast to the content in the diets. This fraction was the fraction that contributed most to the total xanthophyll content, except in the control with a high content of monohydroxy carotenoids (53% of the total xanthophyll content in the yolks). Marigold was the most efficient among the plant supplements tested, despite only a quarter of dihidroxy carotenoids from diets supplemented with 1% and a fifth from diets supplemented with 3% deposited in the yolk.

### 3.2. Egg Yolk Color

Yolks from hens fed the tested diet treatments differed in color parameters determined by both the YCF scale and the CIE Lab color space (Table 4). Yolks from hens fed the control diet had the highest YCF (13.47 vs. 9.13 on average) and a* (18.76 vs. 5.24 on average) values, while L* (66.64 vs. 70.03 on average) and b* (63.08 vs. 68.92 on average) values were lower compared to those of treatment diets. Among treatment diets, hens fed calendula and marigold supplemented diets laid eggs with higher YCF values that were redder (7.81 vs. 2.68), brighter (68.69 vs. 71.37), and less yellow (68.02 vs. 69.82) than those fed dandelion and basil supplemented diets, regardless of supplementation level.

### 3.3. Iron-Induced Lipid Oxidation of Egg Yolks

**Figure 1:**
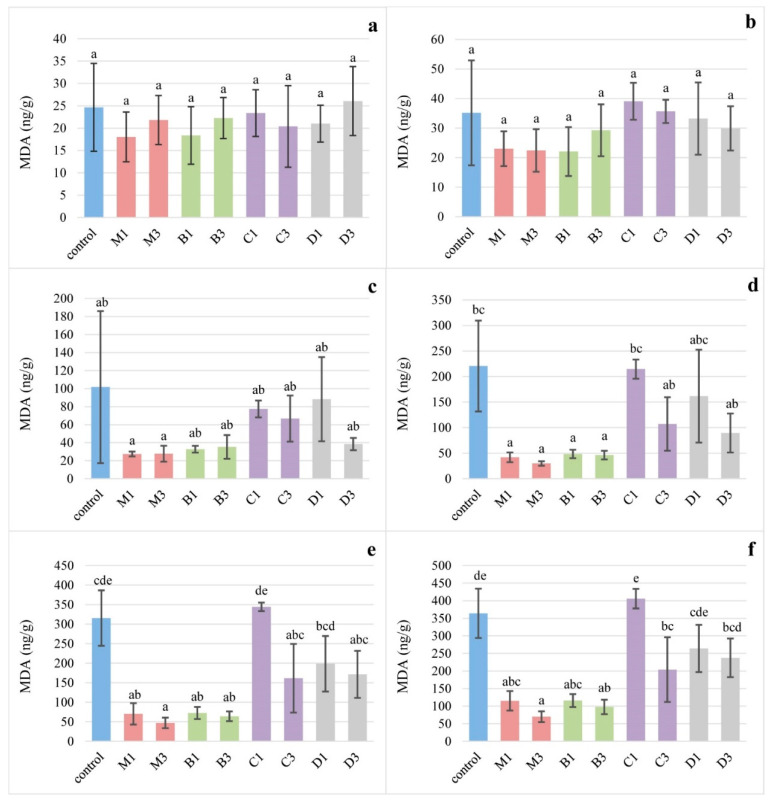
Iron-induced MDA concentration in egg yolks from hens fed diets differentiated in pigment supplementation (B1 and B3—1% and 3% basil herb; C1 and C3—1% and 3% calendula flower; D1 and D3—1% and 3% dandelion flower; and M1 and M3—1% and 3% marigold flower) during incubation from 0 to 250 min ((**a**)—0 min, (**b**)—50 min, (**c**)—100 min, (**d**)—150 min, (**e**)—200 min, (**f**)—250 min). The error bars show the standard deviations for each treatment, and values at each incubation time with different letters differ significantly (*p* < 0.05).

Iron-induced lipid oxidation of egg yolks from experimental and control treatments was monitored during a 250 min incubation at 37 °C, and the results obtained are shown in Figure 1. Pigment sources affected oxidative stability of egg yolks (*p* < 0.0001). During incubation, iron-induced lipid oxidation produced the highest amounts of MDA in the yolks of hens fed the control diet and the 1% calendula supplemented diet (179.22 and 187.95 ng MDA/g on average, respectively). In agreement, these yolks also had the highest values of linear regression slopes of MDA concentration during the incubation period—1.495 and 1.681, respectively. Although lower levels of inclusion of supplemented pigments produced higher amounts of MDA in yolks compared with higher levels of inclusion, this was statistically different (*p* < 0.05) only for diets supplemented with calendula—a 3%-supplementation resulted in 41% lower MDA concentration than a 1%-supplementation after 250 min of incubation. Yolks from hens fed diets supplemented with basil and marigold had the lowest iron-induced MDA concentrations—on average 50.40 and 42.88 ng MDA/g, respectively, and these levels were approximately one-quarter of those found in control yolks during all incubation time points. Consistent with the low values during incubation, the slopes of the linear regression of MDA concentration in the yolks of hens fed diets with 3% of basil and marigold were not significant, while the slopes of MDA concentration in the yolks of hens fed 1%-supplemented diets were the lowest among the other treatments (0.374 and 0.367, respectively).

### 3.4. Sensory Traits of Eggs

Nonparametric statistical analysis of sensory data showed significant differences between treatments in color and flavor of fresh and hard-boiled egg yolk, while significant differences in aroma, texture, and overall acceptability were not found (Table 5). The highest average score for the color of fresh and hard-boiled egg yolk was recorded in the treatment supplemented with 3% of marigold, while the flavor was highest in the control egg yolks. In addition, the color of fresh and hard-boiled eggs from hens fed the control and 3%-marigold supplemented diets was found to be rated significantly higher (*p* < 0.05) compared to treatments with basil, calendula and dandelion. The flavor scores of eggs from the control diet were rated significantly higher than all other treatments, except for supplemented with 1% of marigold.

## 4. Discussion

Diets supplemented with basil herb and flowers of calendula, dandelion, and marigold differed in the carotenoid profile of egg yolks, suggesting that eggs may be a source of different fractions of carotenoids in human diets. Varying levels of carotenoids in the hen diet resulted in variable levels in the egg yolk, but the resulting carotenoid profile among the treatments was more similar than would have been expected given the variation in the diet. Although increasing the supplementation level of the pigment source in the hen diet caused an increase in the total carotenoid content in the yolk, this increase was not as high as in the diets. Total carotene was the carotenoid fraction that was least equivalent to the content in the experimental diets. The content of this fraction in the yolk was probably influenced by the provitamin A activity of these compounds; most of the β-carotene is converted to vitamin A, which significantly reduces the deposition in the yolk [32]. The obtained range of total carotene fraction in the egg yolk of the present study is similar to previous studies, regardless of the content or source in the diet. For example, Kotrbáček et al. [33] reported a similar range of β-carotene content (1.07–2.12 μg/g) when *Chlorella* was supplemented in the hen diet at different supplementation levels.

Similar to β-carotene, other carotenoids such as β-cryptoxanthin could also be converted to vitamin A in poultry, although to varying degrees [34]. Moreover, deposition efficiency varies among carotenoids [10] and generally decreases with higher dietary content [5,9], which contributed to the unexpected xanthophyll profile in the present study. The higher supplementation level of pigment sources in hen diets achieved higher xanthophyll content in egg yolk, but it was not 3-fold higher compared to 1%-supplemented diets. In addition, basil, calendula, and dandelion resulted in similar levels of dihydroxy carotenoids in the yolk at both supplementation levels, regardless of the level in the diets. The diets supplemented with marigold were the only ones that achieved significantly higher levels of dihydroxy carotenoids (approximately 3-fold higher at 1% and 4-fold higher at 3% supplementation levels compared to the other treatments). As a result, egg yolks from hens fed diets that were supplemented with marigold had the highest total carotenoid content and were by far the best source of carotenoids for human diet among the pigment sources tested. Compared to the control yolks, basil, calendula, and dandelion had similar contents when these plants were supplemented at the 3% level. Diets supplemented at 1% achieved lower total carotenoid content than the control diet, indicating low bioavailability of carotenoids from plant parts and supporting the use of plant extracts as a source of pigments for egg yolk [6].

Due to the importance of color, studies related to the pigmentation properties of different carotenoid sources are less focused on increasing the carotenoid content in egg yolk. Therefore, comparison of the results of the present study with previous studies is somewhat difficult, especially in the case of less studied plants. As far as we are aware, only studies with marigold and basil have been conducted to determine carotenoid content in egg yolk. Supplementation of marigold extract at the level of 350 mg/kg in the diet containing 26.5% wheat and 35% maize in the study by Skřivan et al. [12] achieved content of dihydroxy carotenoids comparable to the 3% supplementation of marigold in the present study (29.8 μg/g lutein and 19.2 μg/g zeaxanthin). However, Grčević et al. [35] reported almost twice the lutein content in yolks of hens fed maize-based diets supplemented with marigold flower extract (200 and 400 mg/kg) than the content of dihydroxy carotenoids in the present study, noting that the diets contained an absorption enhancer. While marigold was studied as the sole source of carotenoids, this was not the case for basil. Hammershøj and Steenfeldt [36] used a basal diet containing maize silage supplemented with 1.5 and 3% basil herb; the total carotenoid content obtained in the yolk was higher than in the present study, but similarly, higher supplementation did not achieve a higher content (26.71 and 24.51 μg/g, respectively, compared with 13.75 μg/g at 1% and 20.81 μg/g at 3% supplementation level in the present study).

Egg yolk color is commonly determined by the color scale (YCF), with European consumers preferring a coloration between 9 and 14 but with differences between northern and southern countries—while southern countries prefer intensely colored yolks (11–14) northern countries prefer paler ones (9–10) [9]. In the present study, only eggs from control and marigold supplemented diets achieved values in the acceptable range for southern European consumers. Basil, calendula and dandelion achieved lower YCF values, of which only yolks from hens feed calendula-supplemented diets are acceptable in northern European countries. Results of the present study confirm previous findings of the high pigmentating ability of marigold [5,12,35,37]. Due to its high xanthophyll content, marigold flower is already recognized as a suitable alternative to synthetic pigments in egg yolk production and it is already commercially available [12,35].

Previous studies [5,12,35,37] reported both lower and higher YCF and CIE Lab values compared with the present study, in agreement with the variable carotenoid content in the experimental diets. Altuntaş and Aydin [37] showed that supplementation of 2% marigold flour to the maize-based diet resulted in a significant increase in yolk YCF values from 9.77 in the control group to 10.77 in the experimental group. On the other hand, Grčević et al. [35] reported an increase in YCF value from 13.10 to 14.40 and a* value from 10.74 to 14.14 in diets supplemented with 400 mg/kg marigold flower extract and an added absorption enhancer. In contrast to marigold, studies investigating the effect of inclusion of calendula and dandelion flowers or basil herb in hen diets on yolk color is scarce. Compared to the results of the present study, the a* and b* values of the yolks from hens fed diets supplemented with 1.5 and 3% basil in the study by Hammershøj and Steenfeldt [36] were lower (−2.32 and 55.7 vs. 2.51 and 69.30, respectively). In addition, the authors found that the addition of basil did not improve redness and yellowness compared to a basal diet containing maize silage.

The changes in color values, i.e., the increase in YCF score and redness and the decrease in brightness and yellowness of egg yolks from the experimental treatments in the present study are consistent with the changes in the content of carotenoid fractions in the diets. As seen in the control diets and egg yolks, pigmentation properties of the dominant carotenoids in the diets were the most important factor affecting yolk color. However, higher carotenoid content in the same plant did not correspond to a higher response in yolk coloration. Although changes in redness could be detected using the chromameter for all pigment sources except dandelion, this was not the case for the YCF score, and all experimental diets achieved similar values at both supplementation levels. This observation suggests that a higher supplementation level of the plants evaluated in the study was not necessary in terms of yolk pigmentation.

Egg yolks are known for their high fat content and are therefore susceptible to lipid oxidation. However, hens are able to deposit various antioxidants from diets into their egg yolks that could protect the lipids during egg processing. These antioxidants could be synthetic or derived from natural sources [20,21,28]. Although fresh shell eggs are not readily oxidized even during storage [38], the extent of protective properties during induced lipid oxidation could be evaluated. Indeed, the plants investigated in the present study showed a variable effect on lipid stability during iron-induced lipid oxidation of egg yolk. The control and 1% calendula-supplemented treatments had the highest yolk concentrations of MDA during the incubation period, in which a substantial increase began after 50 min of incubation. The high MDA concentration in the control yolks was in agreement with the results of Galobart et al. [39], who found no antioxidant effect of canthaxanthin. When the experimental treatments were considered, the antioxidant effect was evident even in plants that resulted in lower dietary carotenoid content, such as basil and dandelion. Even more, basil improved the oxidative stability of egg yolks compared to calendula and dandelion (on average 67 and 57% lower MDA yolk concentrations after 250 min incubation). Similar results were shown for other herb plants such as thyme [28], oregano [21] or rosemary [39], due to the presence of different phenolic compounds with high antioxidant properties. Since the basil-supplemented diets had low carotenoid content, the phenolic compounds could be responsible for the antioxidant effect during induced lipid oxidation in the present study.

In addition to basil (on average 71% lower yolk MDA concentrations after 250 min incubation compared to the control), marigold supplementation improved the yolks’ oxidative stability (on average 75% lower compared to the control). The antioxidant effect of marigold is probably due to its high carotenoid content. In terms of MDA concentrations in the yolk, the extent of antioxidant effect of both basil and marigold is comparable to the effect of treatments with 1% supplemented olive leaves or 200 mg/kg α-tocopheryl acetate in hen diet evaluated in a study by Botsoglou et al. [40].

In contrast to the effect on total carotenoid content and redness, yolk oxidative stability improved with increasing supplementation level only in calendula-supplemented diets; 3% supplementation achieved a 41% lower concentration of yolk MDA than 1% after 250 min of incubation. Similar results were found for olive leaves [40] and saffron [20]. This finding suggests that increased supplementation of the evaluated plants, although not having a large effect on color, could significantly improve yolk’s oxidative stability and increase the uptake of antioxidant compounds in the human diet.

The addition of basil, calendula or dandelion had an adverse effect on the sensory color and flavor of fresh and hard-boiled eggs. Yolks from the experimental treatments had sensory color values lower up to 2.52 (fresh) and 1.80 (hard-boiled) compared to the yolks from the control and the yolks with 3% marigold supplemented. These results agree with Hammershøj and Steenfeldt [36] who found that the addition of 1.5 and 3% dried basil has no positive effect on the sensory properties of hard-boiled eggs compared to the maize-based control group. It appears that color was the most discriminatory attribute between treatments, since of the other sensory attributes of hard-boiled yolks, the difference between treatments was found only for flavor; the experimental treatments had scores up to 1.1 lower than the control. The obtained results indicate that the addition of plant material as a source of pigment in hen diet had the greatest effect on yolk color and less on other sensory traits. This result is in agreement with the results reported by Spada et al. [41] who found that the addition of synthetic pigments or annatto (*Bixa orellana* L.) in hen diet significantly improves yolk color but has no effect on the odor or texture of hard-boiled egg yolk compared to diets without supplemented pigments.

Sensory color scores of hard-boiled yolks from the experimental diets were generally rated higher than those of fresh yolks within the same treatment (6.10 vs. 5.51). However, these differences were variable between treatments; while color scores of hard-boiled yolks from hens fed marigold-supplemented diets were higher at 1% supplementation and similar at 3% supplementation, they were higher at both supplementation levels for diets supplemented with dandelion (1.29 at 1% and 0.38 at 3%). These results suggest that the color of fresh yolk cannot be an adequate estimator of the sensory color of the cooked egg, since the surface condition of the product, the physical structure of the pigment support, the particle size, and the homogeneity of colorant distribution are some of many factors that influence the color of the cooked yolk and its perception [42]. Moreover, carotenoids are degraded during cooking and Nimalaratne et al. [43] found that contents of lutein, zeaxanthin and canthaxanthin decrease by 8, 11.6 and 6.6%, respectively, after boiling.

Egg yolks from the control treatment had the highest sensory color scores of fresh and hard-boiled yolks, and only eggs from hens fed diets supplemented with marigold achieved similar values (3% for fresh and both 1% and 3% for hard-boiled yolks). As for flavor, both supplementation levels of calendula, 1% supplementation of marigold and 3% of basil achieved similar but lower scores compared to control egg yolks. However, since the treatment effect was not significant for other sensory traits, these plant supplements should be considered for natural pigmentation of egg yolks. In this regard and in terms of sensory scores, calendula and marigold were the most efficient.

## 5. Conclusions

Supplementation of hen diet with basil, calendula and dandelion did not yield carotenoid content, color, and sensory color of fresh and hard-boiled yolks comparable to commercial pigment or the widely used marigold. The 3% supplementation of hen diet with these plants resulted in similar carotenoid content to the control diet (20.81, 21.76, 22.80 vs. 21.25 µg/g), but lower than marigold flower at 1% supplementation (33.96 µg/g). Only calendula at 3% supplementation reached similar yolk color to the marigold, but only at a lower supplementation level. Both supplementation levels of calendula, dandelion, and basil resulted in similar yolk color, regardless if measured instrumentally or sensory. However, when considering oxidative stability, 3% supplementation decreased MDA production compared to the 1% level. Additional, basil improved the yolk’s oxidative stability (50.40 ng MDA/g) comparably to marigold (42.88 ng MDA/g), regardless of the low carotenoid content. Supplementation of the tested plants decreased the sensory color and flavor of fresh and hard-boiled eggs compared to the control or marigold, but without adverse effects on yolk aroma, texture, and overall acceptability. The 1% supplementation level of basil, calendula and dandelion is sufficient when coloration is targeted, but if added value is desired, 3% supplementation will yield higher carotenoid content and improved oxidative stability. Further assessments of evaluated plants’ suitability should be compared in terms of economic relevance and market research.

## Figures and Tables

**Table 1:** Standard diet feed composition and calculated nutrient composition.

Ingredient	Content (g/kg)
Maize	645.2
Soybean meal	175
Sunflower meal	80
Calcium carbonate	80
Monocalcium phosphate	8
Sodium chloride	5
DL methionine	1.8
Premix ^1^	5
Calculated nutrient composition	
Crude protein	149.1
Crude fat	23
Crude fibre	26
Crude ash	101
Calcium	34.2
Phosphorus	5
Sodium	1.9
Metabolic energy (MJ kg^−1^)	9.87

^1^ The premix provided per kg of diet: Vitamin A 12,000 IU, Vitamin D3 1500 IU, Vitamin E 10 mg, Vitamin K3 2 mg, Vitamin B1 2 mg, Vitamin B2 5 mg, Vitamin B3 30 mg, Vitamin B5 7 mg, Vitamin B6 2 mg, Vitamin B7 0.05 mg, Vitamin B9 0.5 mg, Vitamin B12 12 mg, Vitamin C 15 mg, Choline 500 mg, I 0.5 mg, Fe 60 mg, Cu 3 mg, Mn 80 mg, Zn 50 mg, Co 0.15 mg, Se 0.15 mg.

**Table 2:** The socio-demographic characteristics of the respondents in sensory analysis.

Characteristic	Percentage (%)	Characteristic	Percentage (%)
Gender		Household income	
Female	52.6	Low	6.2
Male	47.4	Middle	59.8
Age (years)		Upper Middle	30.9
<20	9.3	High	3.1
21–30	29.9	Education level	
31–40	26.8	Elementary school	2.1
41–50	20.6	High school	24.7
51–60	6.2	University degree	73.2
>61	7.2		

**Table 3:** Contents of carotenoid compounds (average ± SD) in diets and egg yolks of hens fed diets differentiated in pigment supplementation (B1 and B3—1% and 3% basil herb; C1 and C3—1% and 3% calendula flower; D1 and D3—1% and 3% dandelion flower; and M1 and M3—1% and 3% marigold flower).

Carotenoids	Control	M1	M3	B1	B3	C1	C3	D1	D3	*p*
Diets (µg/g DM)										
Total carotenes	1.08 ± 0.13 ^e^	7.37 ± 0.48 ^c^	12.73 ± 3.29 ^b^	2.55 ± 0.26 ^d,e^	5.15 ± 0.45 ^d^	8.03 ± 0.42 ^c^	17.24 ± 2.34 ^a^	10.67 ± 0.84 ^b^	25.67 ± 3.87 ^a^	<0.0001
Monohydroxy carotenoids	6.12 ± 0.19 ^b^	1.84 ± 0.09 ^c^	1.85 ± 0.06 ^c^	1.67 ± 0.26 ^c^	1.94 ± 0.17 ^c^	5.83 ± 0.43 ^b^	12.35 ± 1.75 ^a^	5.60 ± 1.03 ^b^	11.67 ± 1.12 ^a^	<0.0001
Dihidroxy carotenoids	9.44 ± 0.61 ^f^	106.23 ± 5.35 ^a,b^	299.49 ± 5.46 ^a^	10.04 ± 0.97 ^e,f^	14.14 ± 0.65 ^d^	11.24 ± 0.87 ^e^	18.44 ± 0.72 ^b,c^	8.70 ± 0.27 ^g^	16.17 ± 1.19 ^c,d^	<0.0001
Polioxy carotenoids	nd	nd	nd	nd	nd	3.29 ± 0.93 ^c^	7.69 ± 1.37 ^b^	1.69 ± 0.18 ^d^	15.32 ± 0.86 ^a^	<0.0001
Total xanthophylls	15.40 ± 1.14 ^f^	108.07 ± 5.37 ^a,b^	301.34 ± 5.44 ^a^	12.11 ± 0.55 ^g^	16.97 ± 0.75 ^e^	20.36 ± 1.45 ^d^	38.48 ± 3.13 ^c,d^	15.99 ± 0.75 ^e,f^	43.16 ± 1.92 ^b,c^	<0.0001
Total carotenoids	16.48 ± 1.12 ^f^	115.44 ± 2.82 ^a,b^	314.07 ± 4.78 ^a^	14.66 ± 0.78 ^f^	22.12 ± 1.11 ^e^	28.39 ± 1.86 ^d^	55.72 ± 4.68 ^c^	26.66 ± 1.26 ^d,e^	68.83 ± 5.28 ^b,c^	<0.0001
Egg yolks (µg/g)										
Total carotenes	1.71 ± 0.25 ^a,b,c^	1.59 ± 0.42 ^a,b,c^	1.49 ± 0.47 ^b,c^	1.31 ± 0.18 ^b,c^	1.99 ± 0.33 ^a,b^	1.86 ± 0.21 ^a,b^	2.60 ± 0.38 ^a,b^	1.11 ± 0.19 ^c^	1.62 ± 0.34 ^a,b,c^	<0.0001
Monohydroxy carotenoids	9.30 ± 1.03 ^a^	3.30 ± 0.50 ^b,c^	2.64 ± 0.42 ^c,d^	2.19 ± 0.40 ^d^	2.64 ± 0.31^c,d^	2.15 ± 0.23 ^d^	3.56 ± 0.28 ^a,b,c^	2.19 ± 0.20 ^d^	3.60 ± 0.39 ^a,b^	<0.0001
Dihidroxy carotenoids	10.36 ± 1.00 ^d,e^	29.06 ± 1.32 ^a,b^	62.85 ± 3.81 ^a^	10.02 ± 0.60 ^e^	16.05 ± 0.46 ^b,c^	10.04 ± 0.66 ^e^	13.80 ± 0.80 ^c,d^	9.82 ± 0.29 ^e^	16.47 ± 0.76 ^b,c^	<0.0001
Polioxy carotenoids	nd	nd	nd	nd	nd	1.28 ± 0.53 ^b^	1.95 ± 0.71 ^a,b^	2.59 ± 0.58 ^b^	1.96 ± 0.46 ^a,b^	<0.0001
Total xanthophylls	19.54 ± 1.99 ^c,d^	32.37 ± 1.10 ^a,b^	65.46 ± 3.64 ^a^	12.44 ± 0.95 ^f^	18.82 ± 0.57 ^d^	13.41 ± 0.20 ^e,f^	19.16 ± 1.18 ^d^	14.25 ± 0.37 ^e^	21.18 ± 1.39 ^b,c^	<0.0001
Total carotenoids	21.25 ± 2.10 ^c,d^	33.96 ± 1.28 ^a,b^	66.95 ± 3.56 ^a^	13.75 ± 1.08 ^e^	20.81 ± 0.88 ^d^	15.27 ± 0.27 ^e^	21.76 ± 1.41 ^c,d^	15.36 ± 0.37 ^e^	22.80 ± 0.87 ^b,c^	<0.0001

Values in a row with different letters differ significantly (*p* < 0.05). nd: not detected.

**Table 4:** Egg yolk color (average ± SD) according to the CIE Lab scale and DSM Yolk Color Fan (YCF) from hens differentiated in pigment supplementation (B1 and B3—1% and 3% basil herb; C1 and C3—1% and 3% calendula flower; D1 and D3—1% and 3% dandelion flower; and M1 and M3—1% and 3% marigold flower).

Carotenoids	Control	M1	M3	B1	B3	C1	C3	D1	D3	*p*
CIE										
L*	66.64 ± 0.74 ^f^	68.33 ± 0.88 ^e^	66.43 ± 1.05 ^f^	71.63 ± 1.07 ^a^	71.18 ± 1.88 ^a,b^	70.59 ± 0.79 ^c^	69.42 ± 1.72 ^d^	71.66 ± 1.74 ^a^	71.00 ± 1.28 ^b,c^	<0.0001
a*	18.76 ± 0.92 ^a^	7.28 ± 1.34 ^c^	12.18 ± 1.88 ^b^	2.03 ± 1.51 ^f^	2.99 ± 1.06 ^e^	4.79 ± 1.68 ^d^	7.00 ± 2.90 ^c^	2.55 ± 1.19 ^e,f^	3.13 ± 1.85 ^e^	<0.0001
b*	63.08 ± 0.64 ^f^	68.50 ± 1.06 ^d^	66.80 ± 1.44 ^e^	69.14 ± 1.11 ^c,d^	69.45 ± 2.28 ^b,c^	68.54 ± 0.45 ^c,d^	68.22 ± 1.96 ^d^	70.26 ± 2.08 ^a,b^	70.44 ± 2.02 ^a^	<0.0001
YCF	13.47 ± 0.52 ^a^	10.67 ± 0.72 ^b,c^	11.47 ± 0.83 ^b^	7.67 ± 0.82 ^d^	8.13 ± 0.52 ^d^	9.73 ± 1.33 ^c^	9.73 ± 1.10 ^c^	7.73 ± 0.59 ^d^	7.87 ± 0.52 ^d^	<0.0001

Values in a row with different letters differ significantly (*p* < 0.05).

**Table 5:** Sensory traits (average ± SD) of fresh and hard-boiled egg yolks from hens differentiated in pigment supplementation (B1 and B3—1% and 3% basil herb; C1 and C3—1% and 3% calendula flower; D1 and D3—1% and 3% dandelion flower; and M1 and M3—1% and 3% marigold flower).

Carotenoids	Control	M1	M3	B1	B3	C1	C3	D1	D3	*p*
Fresh yolk										
Sensory color	7.17 ± 2.15 ^a^	5.63 ± 2.09 ^b^	7.28 ± 1.77 ^a^	4.94 ± 2.16 ^b,c^	5.18 ± 1.96 ^b,c^	5.51 ± 1.86 ^b,c^	5.19 ± 1.92 ^b,c^	4.65 ± 2.07 ^c^	5.72 ± 1.79 ^b^	<0.0001
Hard-boiled egg yolk										
Sensory color	7.13 ± 2.08 ^a^	6.58 ± 1.93 ^a,b^	7.28 ± 2.14 ^a^	5.38 ± 2.19 ^b,c^	5.33 ± 1.91 ^c^	6.02 ± 2.16 ^b,c^	6.14 ± 1.88 ^b,c^	5.94 ± 2.21 ^b,c^	6.10 ± 2.01 ^b,c^	<0.0001
Aroma	6.08 ± 2.43	6.09 ± 2.08	5.79 ± 2.56	5.44 ± 2.29	5.37 ± 2.05	5.92 ± 2.18	5.82 ± 2.30	5.56 ± 2.26	5.60 ± 2.22	0.338
Flavor	7.21 ± 1.81 ^a^	6.62 ± 2.06 ^a,b^	6.11 ± 2.33 ^b^	6.12 ± 2.16 ^b^	6.30 ± 2.16 ^a,b^	6.31 ± 1.94 ^a,b^	6.39 ± 1.86 ^a,b^	6.18 ± 1.99 ^b^	6.21 ± 1.96 ^b^	0.035
Texture	7.10 ± 1.76	6.77 ± 1.67	6.46 ± 2.18	6.38 ± 1.92	6.16 ± 1.86	6.62 ± 1.95	6.29 ± 1.91	6.41 ± 1.92	6.37 ± 2.04	0.140
Overall acceptability	6.86 ± 1.74	6.89 ± 1.48	6.40 ± 2.08	6.08 ± 1.83	6.16 ± 1.56	6.31 ± 1.84	6.40 ± 1.75	6.32 ± 1.68	6.18 ± 1.93	0.064

Values in a row with different letters differ significantly (*p* < 0.05).

## Supplementary Materials

The following are available online at https://www.mdpi.com/article/10.3390/foods10040721/s1, Table S1: Contents of carotenoid compounds (average ± SD) in plants part used as pigment sources in hen dites.
