# Supplementary material for: Plant Carotenoids as Pigment Sources in Laying Hen Diets: Effect on Yolk Color, Carotenoid Content, Oxidative Stability and Sensory Properties of Eggs
Source: Foods. 2021 Mar 29;10(4):721. doi: 10.3390/foods10040721 (PMC8066449; doi:10.3390/foods10040721)
Supplement: Supplementary file 1 [file foods-10-00721-s001.pdf]

Table S1. Contents of carotenoid compounds (average ± SD) in plants part used as pigment sources in hen diets.

Carotenoids	Marigold Flower	Basil Herb	Calendula Flower	Dandelion Flower
		μg/kg DM		
Total carotenes	428 ± 21	189 ± 14	679 ± 21	786 ± 23
Monohydroxy carotenoids	nd	8 ± 1.4	353 ± 20	345 ± 21
Dihidroxy carotenoids	9572 ± 141	245 ± 14	322 ± 28	248 ± 16
Polioxy carotenoids	nd	nd	653 ± 24	506 ± 34
Total xanthophylls	9572 ± 141	286 ± 21	1328 ± 75	1099 ± 74
Total carotenoids	10000 ± 121	476 ± 35	2007 ± 96	1885 ± 51

DM: dry matter; nd: not detected.
